# Drag reduction technology and devices for road vehicles - A comprehensive review^☆^

**DOI:** 10.1016/j.heliyon.2024.e33757

**Published:** 2024-07-02

**Authors:** Michael Gerard Connolly, Alojz Ivankovic, Malachy J. O'Rourke

**Affiliations:** School of Mechanical and Materials Engineering, University College Dublin, Belflied, Dublin 4, D04 PR94, Ireland

**Keywords:** Drag reduction, CFD, Aerodynamics, Appendable devices, Vehicle add-ons

## Abstract

This paper addresses the critical role of drag reduction technology in the evolution of road vehicle design amidst the ongoing climate crisis. With transportation accounting for a substantial portion of the EU's greenhouse gas emissions, the shift towards alternatively powered vehicles highlights the need for innovative solutions to extend range, reduce fuel consumption, and lower emissions. This review thoroughly outlines the literature on appendable drag reduction devices, encompassing both passive and active techniques, and their applicability across a variety of road vehicles, including light and heavy-duty transport. Methods applied to simplified bodies such as the Ahmed or other commonly studied generic bluff bodies are clearly distinguished from those applied to more detailed road vehicles, where results hold greater practical significance due to authentic geometry. A combination of both wind tunnel and CFD works are outlined with insights given into how advancements in both computing power and CFD will greatly enhance the future outputs of drag reduction research for road vehicles. Finally, an outlook is provided on the future of the technology and how increased consumer demand for configurable vehicles will encourage increased engagement between drag reduction device manufacturers and automakers to improve the device mounting process.

## Highlights

• Publications related to automotive drag reduction techniques over the last half century are comprehensively reviewed.
• The need for configurable vehicles is described as some users will require low drag modifications.
• Future policy encouraging auto manufacturers to include standardised mounting points for such devices should be considered.

## 1 Introduction

With the climate crisis front and centre, the need for climate action in all sectors is paramount, especially in transport, which is currently responsible for 25% of total EU GHG emissions [1]. With COP 28 having concluded in December of 2023, and all countries, some reluctantly, agreeing to transition away from fossil fuels [2], the potential for alternatively powered transport grows, as increased numbers of electric and hydrogen-powered vehicles are sold each year. With 21.6% of all new car and van registrations in the EU being electric [3], the trends are promising but the electric transition is not without issues. Range anxiety plays out as a major obstacle for a lot of users, particularly those in rural areas who travel long distances [4]. Other barriers can include charging infrastructure, high initial vehicle cost, battery life, limited model availability, and a general lack of knowledge about the new technology can make users hesitant to switch [5]. Fuel costs are another reason to desire reduced fuel consumption. As fuel prices rise, drivers become more aware of the need for better fuel economy and may seek out additional methods to reduce drag on their vehicles. Over 50 years on from the 1973 fuel crisis, and with the wars in the Middle East and Ukraine having the potential to affect fuel prices, it is more important than ever that we seek out novel methods to improve our fuel consumption. Following the 1973 fuel crisis, a focus was placed on reducing drag and what followed was a dramatic change in how vehicles looked in the decades to come, supported with increasing numbers of literature publications on how vehicles could reduce drag using novel devices or appendable technology.

A road vehicle experiences four primary forces in transit, namely, acceleration, drag, rolling resistance, and gradient resistance. The relevant importance of each of these is highly dependent on the driving cycle for a given vehicle. Acceleration losses play a big role where vehicles start and stop frequently whereas drag is the dominant force for constant high-speed driving. Aerodynamic drag can then be decomposed into pressure and skin friction drag, with approximately 90% coming from the pressure component due to a road vehicle's inherently bluff shape [6]. This pressure drag is primarily created by high-pressure frontal stagnation zones and low under-pressured wake regions formed when the flow abruptly separates over the bluff vehicles. For this reason, a vehicle's front and rear faces are subject to large drag forces. Other areas where drag is produced include underbody components, mirrors, wheels, and internal flows. Lift-induced vortex drag is also an issue where large low-pressure vortices form and extract energy from the flow, increasing pressure drag. A car's A and C pillars are locations where strong vortices can form. Based on the global need for drag reduction in the transport sector, all vehicles will need to undergo some form of drag reduction modification. Vehicles such as passenger cars, light-duty vans, heavy-duty articulated trucks, buses, vehicle combinations such as car-trailers, and special vehicles such as police cars, taxis and ambulances all need drag reduction to realise the need for reduced fuel consumption, less GHG emissions, and reduced spending on fuel.

Drag reduction research in the early years was primarily conducted using small-scale wind testing, with a number of large-scale facilities available to few other than automakers and large research institutes. Today the number of large or medium-scale facilities has largely increased while small-scale testing still plays an important role in academic research. Computational Fluid Dynamics (CFD) has over the last 20 years become a driving force for modern drag reduction research, especially in the last 10 years as computing power has vastly improved and the cost of high-performance computing (HPC) equipment is becoming more affordable to a larger audience of universities and companies, including the use of cloud computing resources enabling CFD simulations to be run remotely on pay as you go resources. Both open-source CFD code like OpenFoam and commercial code such as ANSYS Fluent are used to simulate the flow field around a road vehicle and predict the drag and lift forces present on it. Reynolds Averaged Navier Stokes (RANS) simulations have been the industry standard in the past due to their low computational cost enabling quick simulation turnaround and suitability for large run studies in comparison to Large Eddy Simulations (LES) or Hybrid LES. RANS has proven very effective at predicting drag but can struggle to accurately predict lift [7]. Hybrid LES such as IDDES has been shown to have a better correlation with wind tunnel data however the additional computational cost can be many times greater [8]. Machine learning and AI will play an ever-increasing role in the future of drag reduction research as advanced models will be used to both optimise and predict the novel shapes of drag reduction devices that will lead to maximum gains.

The technology and devices currently used to realise drag reduction can be broadly broken up into two subcategories, namely passive and active drag reduction. Passive devices modify the shape of a vehicle without the need for an energy input or active control. Examples include wheel covers, vortex generators, boat tails, spoilers, diffusers, underbody covers, and turning vanes. Active devices are characterised by moving parts and systems that require energy inputs to realise a drag reduction. Such devices include suction or blowing systems, moving flaps and dynamic spoilers. Passive devices are preferred to active solutions as they do not require an energy input and do not require the apparatus needed to provide the energy input. Additionally the less active the components, the less movement of the devices, and the less maintenance required. EU regulations [9] & [10] are in place to ensure the devices conform to maximum vehicle dimensions and are appropriately mounted and used to ensure safety for all road users. The need for drag reduction in the current climate is self-evident. Presented next is a detailed literature review of some of the most significant publications in the field. While not exhaustive, it is comprehensive and will give any newcomer to the field a satisfactory insight into what has been done in the past and what gaps remain to be filled in the future. This article aims to provide an up-to-date review of past and current methods for drag reduction on simplified and detailed bodies, with a more comprehensive review provided for the detailed bodies given their increased relevance and similarity to production road vehicles. Summary statements are given at the end of each section to provide a concise review of the most relevant points within the literature and what future trends are to be expected.

## 2 Literature review

**Figure 1:**
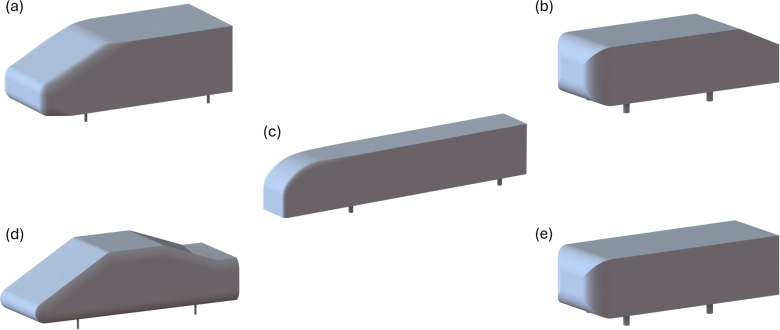
Commonly studied simplified bodies; (a) Windsor Body [11] (b) Slanted Ahmed Body [12] (c) Ground Transport System [13] (d) SAE Notchback Model [14] (e) Square Back Ahmed Body [12][15].

**Figure 2:**
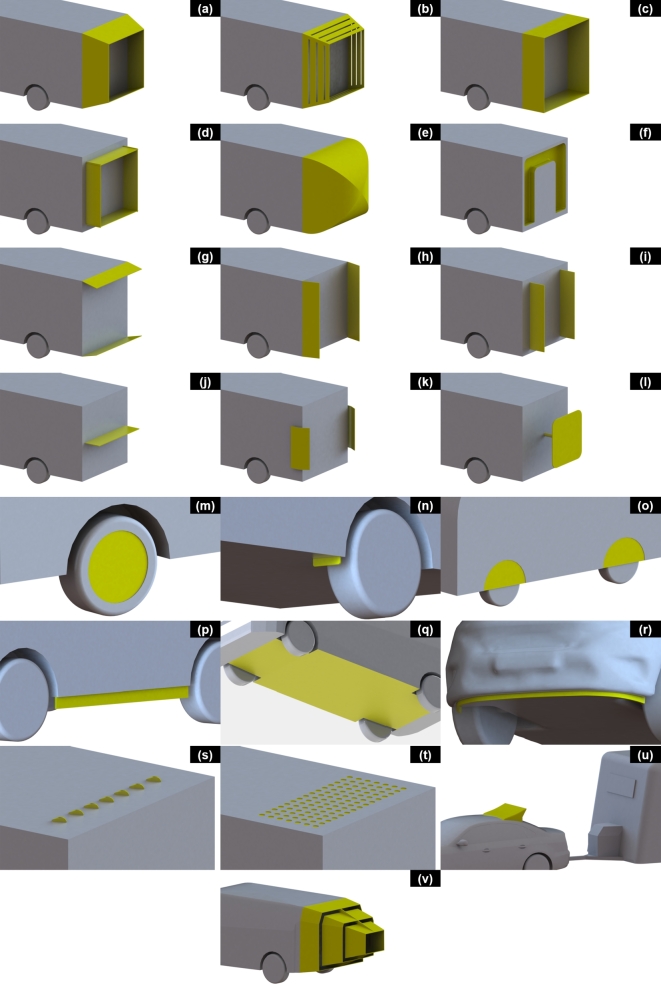
Images of the most commonly referenced passive drag reduction devices described in this article. Table 1 outlines the corresponding name for each device.

The research described hereafter is subdivided into methods applied to simplified and realistic bodies. The simplified bodies are then further categorised as active and passive, whereas the realistic bodies are separated according to their vehicle type. The margin between what is a simplified body and what is an adequately detailed realistic body is not always clearly defined, therefore in some categorisations, author discretion was applied. Fig. 1 demonstrates some of the commonly studied simplified bodies. Additionally, Fig. 2 is provided to outline some of the visual features on the most commonly referenced devices.

### 2.1 Simplified bodies

#### 2.1.1 Active drag reduction

Li et al. [16] performed wind tunnel testing on an Ahmed body at 5° yaw with blowing applied at the windward trailing edge using fluid jets of both high and low frequency to reduce drag by 7%. The blowing helped control the size and intensity of the wake while reducing turbulent kinetic energy. The bi-frequency approach was the most efficient blowing method for reducing drag when accounting for the required energy input. Hoffmann et al. [17] studied a full boat tail cavity combined with fluid oscillators and vortex generators (VGs) to increase the maximum effective angle of the boat tail on a simple square-back model. The optimal using just the boat tail was a 33% drag reduction angled at 15°. Using the VGs, angles past 15° could still give appreciable drag reduction but not as high as the optimal 33%, meaning the best option was to use the tail correctly angled without other components. Barros et al. [18] added pulsed jets and a Coanda surface/flap to the rear of a square-back Ahmed body to reduce drag. Variable frequency blowing was found to lower wake turbulent kinetic energy and increase base pressure. Low-frequency blowing reduced drag by 10% by reducing wake size and enhancing momentum entering the wake, while high-frequency blowing also reduced drag by 10% but did so by lengthening the wake and reducing momentum entrainment. Adding the Coanda surface with the blowing produced a 20% drag reduction. Howell et al. [19] performed wind tunnel testing on a simple square-back vehicle with no wheels or ground simulation to reduce drag using a combination of rear cavities and rear blowing. The drag reduction was dependent on the bleed rate, for which higher rates had higher drag reductions. The majority of the reduction was due to the presence of the cavity alone, with the bleeding only adding to it slightly. It was concluded that the power requirement for the bleeding could offset or negate the additional savings. Yurchenko [20] detailed the potential to reduce drag on an angled aerofoil using small heated riblets that interact with the flow. Little information was provided on the drag reduction mechanisms. The angled aerofoil could be compared to the rear windshield of a fastback vehicle, hence it would be of interest to attempt something like this on a ground vehicle to see if the drag could be reduced. Zhang et al. [21] investigated active drag reduction on a 25-degree Ahmed body by applying steady blowing on the body's rear edges. Four individual blowing sections/actuations produced drag reductions of between 6-14% when used individually, but when combined in an optimal configuration, produced an appreciable 29% reduction on the baseline body. The reductions were a result of modifying the wake's shape and size, resulting in significant base pressure increases. Geropp and Odenthal [22] studied a 2D car in a wind tunnel that incorporated rear blowing at the top and bottom of the base combined with a Coanda effect to eliminate the vehicle's rear wake. The base pressure was found to rise by 50% which equated to a 10% drag reduction. In reality, the results in 2D might not apply directly to an actual 3D car. This is compounded by the challenges related to the energy input for the system and the extensive modifications necessary for efficient operation. Gagnon et al. [23] explored a novel approach to reduce drag and recapture some flow energy by placing a rotating paddle wheel in the wake of a slanted Ahmed body. 2D CFD simulations found that the paddle wheel was able to capture an appreciable amount of swirling energy from the wake while reducing drag by 6.9%. If the wheel was powered, then the reduction could rise to 7.6%. The drag reduction mechanisms included reducing wake turbulent kinetic energy and weakening the wake vortices, resulting in increased base pressure.

The general trend is that active drag reduction techniques are usually used in conjunction with a passive device such as a rear cavity or slanted extension. Most of the time, the majority of the benefit can come from just the passive device's contribution, therefore reducing the relative benefit of adding the active device when considering the required energy input and added infrastructural overhead for fitting the device. This is why passive techniques are more likely to succeed in terms of future implementation on road vehicles.

**Table 1:** Commonly used names for the devices outlined in Fig. 2.

	Device Name		Device Name
(a)	Angled Cavity	(l)	Rear Offset Plate
(b)	Ventilated Angled Cavity	(m)	Wheel Covers
(c)	Straight Cavity	(n)	Wheel Deflectors
(d)	Inset Cavity	(o)	Wheel Spats
(e)	Filled Boat Tail	(p)	Side Air Dams
(f)	Indented Vortex Trap	(q)	Underbody Tray Cover
(g)	Rear Horizontal Angled Flaps	(r)	Front Underside Spoiler
(h)	Rear Vertical Angled Flaps	(s)	Vortex Generators
(i)	Rear Vertical Slats	(t)	Textured Panels
(j)	Rear Horizontal Slat	(u)	Tow Vehicle Roof Deflector
(k)	Rear Turning Vanes	(v)	Multi Stage Converging Cavity

#### 2.1.2 Passive drag reduction

Lorite-Díez et al. [24] outlined a drag reduction study on a square-back Ahmed body in which different rear cavities were studied. It found that an interesting and novel-shaped rear cavity outperformed a straight cavity, especially in crossflow, offering a 10% reduction in yaw. Additionally, the straight cavity's performance decreased with increasing yaw angle. Testing was done on a very small-scale model in a small wind tunnel. Garcia De La Cruz et al. [25] used adjustable base flaps of length and angle to optimise the drag reduction on the Ahmed body in a crosswind. It found that two rear flaps configured non-symmetrically were optimal and that using adaptable flaps in a crosswind can improve the reduction by up to 70% when compared to static flaps optimised for direct flow. Wong and Mair [26] investigated how a four-slant full boat tail compared to a two-slant (top and bottom) tail when fitted to the rear of a long, simplified, square section body. Using wind tunnel testing the four-slant variant considerably performed better for a large range of slant angles. This older article outlined no flow visualisation, with little validation and primarily focused on force measurements. Lorite-Díez et al. [27] optimised a rear cavity using an adjoint solver for a long slender blunt body, and found the optimal cavity used a novel curved flap that curved inwards toward the cavity. The flaps were fitted to the top and bottom trailing edges and produced a 43.9% drag reduction by displacing the recirculating zone further downstream. The geometry studied was possibly too simplistic, detracting from the result's relevance to realistic road vehicles. Gilliéron and Kourta [28] performed small-scale wind tunnel testing on an Ahmed body fitted with a vertical square plate placed downstream of the rear to reduce drag. The plate was parallel to the body's rear and a 12% drag reduction was achieved using optimal spacing between plate and rear. The plate manipulates the rear flow of the vehicle, reducing wake intensity. Interestingly, the article further claimed a drag reduction for a similar plate mounted upstream of the Ahmed body, which was surprising, as having a blunt front would most likely increase the combination's drag. Park et al. [29] took an extruded 2D profile with a blunt rear and added rectangular tabs to the trailing edges that projected directly into the flow. A 33% base pressure increase was found due to the best-shaped tabs introducing mismatched vortex shedding into the wake which reduced overall vortex strength in the wake. The main issue with the study was that testing was done at a very low Re on a simplified model. Hassaan et al. [30] carried out CFD testing on a long slender square-back body fitted with a boat tail. The body had no wheels or ground simulation and was a simplified representation of a heavy vehicle. The most effective tail reduced drag by 50% using a 15° slant angle. Larger angles performed worse in direct flow but offered appreciable drag reduction in a crosswind by helping restore the quasi-symmetry in the near wake. Howell et al. [31] investigated the effects of a ventilated cavity on a square-back Ahmed body with variable depth. The straight cavity was ventilated using slots along the cavity's wall. The unventilated cavity performed the best at reducing drag, with better performance for greater depth. When ventilated, the drag reduction was reduced as the cavity depth increased. Hence the slots were seen as a source of drag on the body. It was shown that by modifying the geometry of the slots the drag reduction could be improved. Hirst et al. [32] proposed a new drag reduction device known as the jet boat tail. The device was fitted at the end of a bluff body and captured the freestream flow to form a high-speed jet that was then angled in toward the base of the body, giving a boat tail effect. The high-energy jet entrained freestream flow and increased the overall base pressure on the rear. Using wind tunnel particle image velocimetry (PIV) and a numerical large eddy simulation (LES), a 15% drag reduction was quoted. Pujals et al. [33] took the 25° slanted Ahmed body and outfitted it with an array of cylinders ahead of the slant to generate long streaks of streamwise vortices. The cylinders used were large in comparison to other VG methods sized according to boundary layer height. Using an optimal cylinder array, drag reductions of 10% were reported. PIV and static pressure measurements were used to confirm that the streaks suppressed the rear separation on the 25° slant. Darabasz et al. [34] looked at the flat back Ahmed body with an added rear cavity that included top cavity wall modifications. Notches, imprints, and holes were all studied as possible top-wall modifications. The basic cavity provided a 9% drag reduction, which was increased to 11.5% using the best wall modification of a large imprint or two smaller central imprints. The provision of enhanced wake stability was the main drag reduction mechanism. Beaudoin and Aider [35] took an Ahmed body with a 30° slant and fitted flaps to all its trailing edges to reduce drag. By optimising the angles of the flaps in a wind tunnel, it was found that a 25% drag reduction could be realised. The most effective flaps were those fitted to the C-pillars (17.6% reduction) and the flap on the roof's edge (15%). The flaps on the base area were less effective but still reduced drag noticeably. The main mechanism for drag reduction was the prevention of the wrapping longitudinal vortices that usually form on the slant, which reduces the induced drag and lift penalty. Kowata et al. [36] looked at reducing drag on a square back Ahmed body by adding an underbody slant and rear flaps. Using small-scale wind tunnel testing the slant/diffuser was found to increase drag past an angle of 3° due to underbody flow acceleration reducing pressure and the formation of stronger underbody vortices. Using just a top flap was the least effective for drag reduction. Using a combination of top and side flaps improved performance while using a full rear enclosure offered the best saving for small slant angles. Verzicco et al. [37] outlined an LES study to reduce drag on a square back Ahmed-styled body by fitting a solid boat tail and a square inset cavity. The LES described had 10 million grid points which was significant for the time of publication in 2002. The boat tail was found to reduce drag by 31% outperforming the inset cavity, which only reduced drag by 18%. The drag reduction mechanisms for both devices included increasing base pressure and reducing the intensity of the flow separation. Robertson et al. [38] looked at a simple square back Windsor model with no wheels or ground simulation to investigate the potential drag reduction when incorporating horizontal slats that slightly protruded the rear base of the vehicle. Two configurations were tested, one with 3 slats, and the other with 4 slats, all located on the lower half of the base. Small drag reductions of between 3-3.5% were observed when the underside of the model was smooth. When the model's underside was roughened to represent that of a real car, the drag reductions disappeared and drag increases were reported. This highlights that research published on very simplified smooth bodies will have discrepancies with real road vehicles. Siddiqui and Agelin-Chaab [39] modified the rear of the standard Ahmed body by reshaping it into an elliptical rear, resulting in reduced drag from a less intense rear wake. The issue with the study, however, is the Reynolds number for the work was around 5x10^4^, which is at least an order of magnitude lower than that for full-scale road vehicles travelling at highway speeds. Low Re studies have the issue of enhanced laminar effects which can affect the separation phenomena occurring at the rear of the vehicle, which can lead to results that are not completely comparable to full-scale road vehicles. Aranha et al. [40] detailed how machine learning can be used to generate passive drag reduction devices for the 35° Ahmed body. A sophisticated genetic algorithm was used to design a unique roof flap and diffuser that both increased downforce and reduced drag. The optimised devices were organically shaped such that the flap used extensive curvature and splines while the diffuser had a distinct wavy surface. The flap and diffuser were found to reduce drag by 6.3% and 5.4% respectively. Together they reduced drag by 7.4%. Highlighting that in future years, aerodynamic design may be largely AI-assisted. Khalighi et al. [41] looked at a square back shaped Ahmed body using CFD outfitted with two rear drag reduction devices. The first was an inset cavity that reduced drag by 18%, while the second was a solid-filled boat tail device that reduced drag by 30%. CFD and wind tunnel testing showed similar trends with discrepancies in the absolute drag values. Both devices were found to reduce wake unsteadiness, improve pressure recovery, and reduce turbulence intensities in the wake. Aider et al. [42] studied a modified Ahmed body in which the rear was fully curved and not slanted, similar to a VW Beetle. A line of non-conventional trapezoidal VGs were placed near the region of separation to reduce drag. The maximum reduction was found to be 12%. Having the VGs mounted on a motor allowed them to have their angle adjusted and optimised, yielding a 14% reduction. The VGs caused early separation, producing a larger recirculation bubble with weaker streamwise vortices that drew less energy from the flow. Lajos [43] took a baseline high drag bluff body cuboid and added a corner step/notch to its front face to produce a separation bubble that provided thrust to the body along with helping the flow turn around the cuboid, similar to a radiused edge. Drag reductions of over 50% were quoted from small-scale wind tunnel tests with similar results found when using a fence/forward-facing panel to produce the same effect.

The results outlined for simplified bodies such as percentage drag reductions must be considered only as approximate indicators for performance. All future works that use a simplified body to design/optimise a drag reduction device must ensure they include a study of how it performs on a real detailed vehicle under true road conditions, providing then a comparison between the reductions on the detailed vehicle versus that of the simplified body. For example, the effects resulting from the inclusion of wheels, a moving ground, and underside components can have a huge impact on the results for a real body over a simplified body. Rear cavities are generally the best device for maximising drag reductions on a simplified bluff body. A further improvement is found when the cavity is optimised for crosswind conditions, such as ensuring a minimal wind-averaged drag coefficient.

### 2.2 Realistic bodies

#### 2.2.1 Light duty transport

Capone and Romano [44] carried out wind tunnel testing on a small square-back car fitted with rear horizontal and vertical deflectors. The greatest drag reduction was achieved with deflectors angled 20° inwards, which reduced wake size and reduced wake oscillations. No ground simulation was performed. Nabutola and Boetcher [45] outlined the advantages of air-jet wheel deflectors over the conventional flap deflector. Using a 45° air jet ahead of the front wheels produced a 1.5% drag reduction. Air deflectors have the advantage of not adding a form drag component associated with the deflector itself. All the work was CFD-based using smooth wheels. Ferraris et al. [46] took a small city car and applied flow control devices to the wheel arches that provided air blowing and relief. The devices consisted of front air curtains combined with front and rear blowing slots. Only a 1% drag improvement was realised but the baseline car was already highly optimised, hence added reduction was difficult to find. High-quality CFD and wind tunnel testing was conducted, with the authors outlining some discrepancies between the physical and virtual results. Yang et al. [47] took a generic minivan and outfitted it with a non-smooth roof surface panel to reduce drag. Different patterns were studied such as rectangular and circular concaves depressed into the panel. Following optimisation, the design was seen to offer a 7.7% drag saving as the depressions altered the minivan's wake by thickening the boundary layer and reducing wake intensity after separation. Urquhart et al. [48] optimised a base cavity for a generic square-back vehicle by adjusting the taper on the cavity to provide the best drag reduction in a crosswind. The tapered cavity outperformed the straight cavity, realising a 17.6% vs. 7.5% drag reduction in direct flow. The optimised cavity reduced wind-averaged drag by 14.7% by reducing wake unsteadiness and increasing base pressure. Datta et al. [49] discussed the application of rear fairings, rear flaps, and VGs on a very simplistic car model and reported substantial drag reductions. The low quality of both the CFD and CAD might deem the results untrustworthy, highlighting how some of the CFD work in the literature on drag reduction technology can be questionable. Selvaraju and Satish Kumar [50] added an adapted rear diffuser and VG to a generic SUV. The VG reduced drag by 7% which was high based on other findings for VGs in the literature. When combined with the diffuser the reduction increased to 8%, highlighting the marginal effectiveness of the diffuser design used in the study. The quality of both the CAD and CFD were low compared to modern standards. Irving Brown et al. [51] explored the benefits of incorporating a rear cavity along with passive base bleeding on a full-scale SUV. A recessed body cavity combined with an external cavity produced a maximum of 3.3% drag reduction, with enhanced savings the greater the recess. The addition of passive base bleeding only offered an additional 1% drag saving, noting that it would not be worth implementing for such a small benefit. It would have been interesting to have tested a more optimised base cavity with tapers to see if the reductions could be improved on as the reductions presented were quite low. Vdovin et al. [52] described how the ventilation drag component on the wheels of a passenger car can be altered using different rim designs. 14 different 17-inch rims were studied in a wind tunnel facility on a special rig in the presence of the vehicle and the wheel housing. The power requirement to rotate the wheels was related to the ventilation drag. Interestingly, a fully covered rim did not have the lowest ventilation drag. A rim with a covered outer layer and small interior slots had the lowest, as it enabled any air build-up inside the rim to vent out. Varney et al. [53] studied a generic SUV in which side and roof slots were made towards the rear of the model to provide a combined rear tapering and passive ventilation effect. The tapering was found to offer significant drag reduction, whereas adding the passive ventilation did reduce drag further but by a much smaller amount. The optimal shape consisted of 15° side tapers with a 10° roof taper, which led to significant base pressure increases. De Souza et al. [54] examined the effects of well-accepted drag reduction techniques such as ride height adjustment, underbody treatment, grille shutters, and wheel curtains on a variety of passenger vehicles. Full-scale high-quality wind tunnel testing was performed in which underbody panels were found to be highly beneficial for reducing underbody drag and helped reduce drag elsewhere due to improved underbody flow. Blocking wheel curtains increased drag by 2.1% on a compact sedan, while removing side mirrors reduced drag by 2.4-3.9% depending on the vehicle type. Kim et al. [55] outlined a novel-shaped rear spoiler for a minivan that reduced drag by 5% through diverting flow into the wake and increasing base pressure. The CFD study had just 2 million cells which was far below that used today in best-practice automotive CFD. Paul et al. [56] performed 1:15 scale wind tunnel testing on a generic small car with no ground simulation or rotating wheels. It found that by using an optimal combination of vortex generators (VGs) placed in an array along the vehicle, drag could be reduced by 23%. This reduction was unrealistically high in comparison to other works using VGs. Howell et al. [57] considered how drag can be reduced on a generic square back body using an elongated streamlined tail. Large-scale wind tunnel testing found that including side tapers on the tail increased drag reduction by 60%, while greatly improving the crosswind performance. The tail reduced drag by enabling a smooth pressure increase as the flow transversed the tail. By cutting off half the tail it was found that the device still maintained 83% of its total drag reduction. Nath et al. [58] performed a CFD study on a generic race car in which a drag reduction of 16.5% was found when fitted with a small spoiler and 12° diffuser. The primary drag mechanism was described as reducing the recirculation zone behind the vehicle. Additionally, adding a racing wing was found to increase drag by nearly 40%. Overall, the CFD quality was low with only 1 million cells used to model the race car and its entire domain, with little post-processing of how the drag reductions were produced. Hoerner [59] detailed a wide variety of early wind tunnel testing on passenger cars and provided insights into the drag effects of open windows, shape design, cooling drag, wheel openings, and underside covers. The vehicle geometries discussed in the book are perhaps outdated, for example, it is suggested that by having a fully smooth underside the drag coefficient of a car could drop from 0.6 to 0.3. Ilea and Iozsa [60] analysed how drag was affected as the rear wheels of the vehicle were progressively closed off using CFD. The best drag reduction came from a fully enclosed rear wheel. Additionally, it was found that the optimal height for a rear wheel deflector was not the maximum height studied and that by using a profiled deflector an enhanced drag reduction could be realised but at the expense of taking up added space. Fukuda et al. [61] outlined work by Mitsubishi Motors in which a new car rear spoiler was designed that reduced drag, opposite to most conventional spoilers at the time which increased it. Testing was performed on an Ahmed body, then a small-scale car model, and finally a full-scale car. The new optimised spoiler, shaped like a trigonal pyramid, reduced drag on both a fastback and notchback car by increasing the vehicle's base pressure. The new spoiler however did not provide as much downforce as its conventional alternative. Koike et al. [62] outlined the design process of the VGs fitted to the Mitsubishi Lancer. The optimal location for the VGs was 100 mm ahead of the rear roof separation point. The optimal VG height was found to be 30 mm, which was the height of the boundary layer at their fitted location. Two types of VG were studied namely, a bump and delta wing shape. The delta wing VGs performed best due to the stronger vortices they induced and their inherently smaller projected frontal area. The VGs reduced drag by limiting the drag component associated with the rear roof separation on the Lancer. Urquhart and Sebben [63] studied a full-scale Volvo XC40 fitted with a base cavity of 150-200 mm which was then further extended by 80 mm using adjustable flaps controlled by servos. In direct flow, straight flaps reduced drag by 20%, but when subjected to yaw the reductions fell substantially. By adjusting the flap angles, the cavity was optimised using a surrogate model to perform best as per wind-averaged drag conditions, which offered a 1% improvement over the conventional straight cavity. Urquhart et al. [64] performed a high-fidelity CFD study on a Volvo SUV fitted with 150 mm long rear extensions. Two types of extension were studied, one with a straight extension, and one which kicks out as it extends. In direct flow, the straight extension performed over three times better at reducing drag than the kick-out version. However, when subjected to crossflow, the kick-out extension outperformed the straight one, highlighting the need for devices optimised for wind-averaged drag. Sebben et al. [65] and Sterken et al. [66] similarly discussed how rear extensions on a Volvo SUV can be used to reduce drag by 10-20 counts depending on extension length and design.

Kang et al. [67] described a CFD study on an active rear diffuser that deployed once the car reached a high speed. The diffuser extended out from the base of the car and reduced drag by not allowing the low-pressure underflow to curl up onto the vehicle's base. Seven different variants were studied, with the best having a 450 mm elliptical shape and an average drag saving of 4.2%. When fitted to a YF Sonata production car the device offered an appreciable 5.2% drag reduction. The domain sizing for the CFD study was quite small which could have led to errors in the reported force coefficient. Cho et al. [68] performed a CFD study on a detailed YF Sonata Sedan outfitted with three underside drag reduction devices. The devices included a smooth undercover, underside fins, and L-shaped side air dams. The undercover reduced drag by 8.4% while the other two devices reported slight drag increases, yet when all three were used together an 8.9% reduction was realised due to interaction effects. The primary drag reduction mechanism was outlined as the attenuation of the rear longitudinal vortex pair by enhancing the momentum of the underside flow being up-washed. Cho et al. [69] detailed a study in which plates were added to the rear of a notchback DrivAer model. A horizontal plate was added to the upper lip of the trunk while vertical plates were added to both the trunk and the C pillars. A basic 150 mm horizontal plate offered the best drag reduction of 5.1% by preventing the downwashed flow decreasing the base pressure of the vehicle. For the vertical plates, a 3.6% drag reduction was realised with a wedge-shaped plate on the C-pillar and a square-shaped plate on the trunk lid. This reduced drag by limiting the wrapping side flow and reducing the strength of the C-pillar vortex. Morelli [70] outlined a novel device known as the fluid tail, which consisted of a fan mounted within the rear wheel arches that sucked air from around the wheels and blew it into the rear wake of the vehicle, which was fitted with a flush boat tail cavity of appreciable depth. Experiments on a Fiat Punto 55 reported drag reductions of 18-20%. The suction improved the flow field around the wheels and additionally reduced rear soiling on the hatchback. The device demonstrated well the effectiveness of cavities and base blowing on real square back vehicles. Peterson [71] detailed work carried out by NASA on a box-shaped van fitted with a long solid boat tail along with a truncated solid boat tail. The longer tail was cut/truncated at the correct location based on road tuft testing. Using coast-down testing, the full tail reduced vehicle drag by 32% while the truncated tail reduced it by 31%, highlighting the effectiveness of the tail's shape and curvature. The van used had a well-rounded front with a smooth underside, making its baseline rear drag component high relative to total vehicle drag. McNamara et al. [72] worked on the design of an inflatable boat tail for a passenger vehicle. Two prototype tails were tested on a generic bluff body in which the more convergent tail was found to reduce drag by 82.6% compared to 29.4% for the less convergent tail. The article further discussed the issues with implementing such a device on a real vehicle such as air leakage, appropriate structural design and material selection. Such a device may be more practical in the future with the advent of advanced smart materials for appendable devices. Chen and Khalighi [73] used CFD to predict the drag reductions on a highly detailed pickup truck when appending a boat tail made of plates, a mid-plate attached to the outer tailgate face, and a partial bed cover that extended from the top of the tailgate. The most effective part of the boat tail was the top panel, which at full length could reduce drag by 10 counts. When combined with the partial bed cover, the total reduction reached 21 counts. Drag reduction mechanisms included reducing the pressure on the inside of the tailgate while increasing pressure on its outside. Taniguchi et al. [74] outlined work by Nissan to reduce the drag on their production pickup truck by 12%. A tailgate spoiler was used to help catch the flow that detached from the roof of the pickup at different speeds, reducing drag. Other devices included a front underside spoiler, deflectors fitted between the cabin and bed, and deflectors fitted in the rear wheel housings. The deflectors blocked the flow from taking a path that caused added drag on the frame/bed of the pickup, while the underside spoiler reduced drag on underside components. Sirenko et al. [75] used wind tunnel testing to evaluate the effectiveness of appendable devices on a generic SUV. A rear plate mounted on a strut offset from, and parallel to, the base of the vehicle reduced drag by 6.5%. The greater the plate offset, the greater the drag reduction until a certain point, after which it levelled off. The plate's diameter matched that of a wheel's diameter. The most effective device was a solid boat tail split into slices so that its length could be varied. The full-length tail reduced drag by 26%. With half the tail cut off, the drag reduction only fell to 22.6%. Mixed results were found for VGs, as slight drag increases or reductions were reported depending on configuration. Ohno and Kohri [76] outlined work by Mitsubishi Motors in which side air dams for passenger cars were optimised for drag and lift reduction. In direct flow, it was found that the dams can reduce $C_{D}$ by up to 0.01 as the dam improves the pressure distribution on the rear wheel while enabling underflow acceleration, improving downforce. However, in yaw, they can increase lift as they prevent the side flow from exiting, facilitating a pressure increase underneath the car. A common reason for their scant use on production cars today is that ground clearance issues are of greater concern than minor drag and lift reductions. Hucho et al. [77] detailed how making small geometry adjustments to road cars can reduce drag. Examples of the changes include front-end redesign and nose cone shape optimisation, A and C pillar optimisation, front underside spoiler design, and rear-end redesign. Removing the rain gutters on the A pillars was seen to reduce drag by 7%. Reference was made to how slant angles of 25-30° on fastbacks can result in drag spikes as a result of the well-studied drag crisis. Correct spoiler design was identified as a useful way to reduce drag on a fastback car. While some of the methods discussed are outdated by modern vehicle design, the priority today is still for small geometry modifications rather than invasive changes, such as adding rear cavities. Pamadi [78] described work by NASA on a novel drag reduction technique in which strakes were fitted to passenger cars that ran along the bumper and up the windscreen. The strakes suppressed vortex shedding at the vehicle front and around the A-pillar while producing a front separation bubble that provided thrust due to its low pressure on front surfaces. Testing was performed on a car and van in which the drag reductions were 6.5% and 8.5% respectively at 55 mph. The strakes could be made transparent and retractable to facilitate their implementation as an appendable device. Maxwell et al. [79] discussed two drag reduction devices for a pickup truck in which a small extension wing was attached to the rear of the cab to help guide the flow, along with a partial bed cover. The wing was found to reduce drag by 5% while the cover reduced it by 6%. Together they produced a 17% drag reduction along with an additional lift reduction. The devices improve the pressure distribution on the rear surfaces of the tailgate and cab to facilitate drag reduction. Carr [80] detailed large-scale wind tunnel and on-road testing of a hatchback fitted with various devices to reduce drag by 30%. The most effective were a front air dam (11.1%) and a rear roof spoiler (5-9%). The rear roof spoiler reduced drag by preventing flow reattachment on the rear roof which came at the cost of added rear soiling. Less effective devices included turning vanes at the A-pillars to help guide the flow around the pillar (3%). The vanes must be kept small, as larger vanes were found to increase drag. Wheel cap covers and rear wheel spats reduced drag by only 0.4% and 2.2% respectively. Buchheim et al. [81] reviewed work carried out to reduce the drag coefficient of the contemporary passenger car and how future cars could have reduced coefficients of between 25-35% when using optimal styling without the need for obtrusive devices. Examples included adjusting roof camber, windshield angle, front end curvature, rear end design, rear glass angles, and underbody design with optimum diffuser angles. Much of what was discussed is implemented today on low-drag passenger cars.

**Figure 3:**
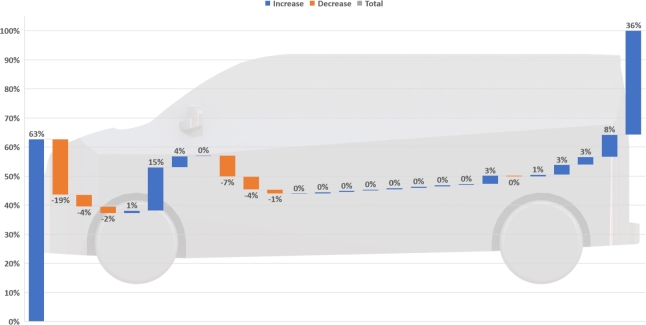
Accumulated drag distribution for a generic light-duty van. Note that the rear is responsible for nearly 40% of the total vehicle drag, outlining the need for rear drag reduction devices for this type of vehicle.

In summary, small modifications such as altering the surface roughness of a vehicle with depressions or bumps, generally offers relatively small reductions even when optimised. Vortex generators (VGs) are a well studied add-on and have been shown to provide mixed results. Realistic drag reductions for optimised VGs are at most approximately 5%. Similar to the simplified bodies, a rear cavity device is found to perform the best in reducing drag on a realistic light-duty vehicle. Fig. 3 outlines the suitability and need of light-duty transport for rear drag reduction devices. Smoothening the underside using an undertray nearly always offers an appreciable drag reduction. Additionally, a smooth underside can increase the effectiveness of a rear cavity, hence demonstrating that the interference effects between devices are crucial to consider when maximising overall gains.

#### 2.2.2 Heavy duty transport

Wood and Bauer [82] outlined three novel devices to improve fuel economy for tractor-trailers. These included a vortex trap device for within the combination gap, a vortex strake device fitted to the rear of the trailer, and an undercarriage flow device. Tested on the road they improved fuel economy by 10% from a reported 30% drag reduction. The vortex strake device which consisted of protruding strips is not seen today as it may not reduce drag as reported or adds an operational overhead, such as when reversing into tight loading bays. Cooper [83] looked at the history and contemporary usage of boat tails, side skirts, gap fillers, and roof deflectors on tractor-trailers. Outlining that together they can save 4,000 gallons of fuel per 125,000 miles. It highlighted that trailer-fitted devices have a slower payback time compared to tractor devices as there are multiple trailers for any one tractor, therefore a trailer can spend considerable idle time in the yard over its lifetime. Englar [84] took a very simplified small-scale truck model and added blowing to the leading and trailing edges of the trailer, realising a near 50% drag reduction over the baseline vehicle. This article used many model simplifications and reported an unusually high reduction for an active blowing technique. Van Raemdonck and Van Tooren [85] detailed the results from high-quality wind tunnel and on-road testing of side skirts and boat tails fitted to tractor-trailers. The patented side-skirt system reduced drag by 17% while the optimal boat tail offered a 12% reduction. The optimal boat tail angle was 10°, with the longer the tail the greater the saving. Road tests showed a 7.5%, 6% and 3% fuel economy improvement for the 2 m, 1.5 m and 1 m boat tails. Removing the bottom panel of the boat tail more than halved the reductions. Bayindirli [86] proposed a new type of drag reduction device for a bus that consisted of a half-cylinder rod placed in front of the bus. After testing different diameter cylinders in a wind tunnel, the best configuration realised a 10% drag reduction. The device had the advantage of being hinge-mounted, enabling movement between the deployed and un-deployed positions. Yang and Ma [87] detailed drag reduction on a generic truck fitted with a roof fairing and a combination of cylinders attached to the base of the truck. Using relatively low-quality CAD and CFD, the authors outline how the fairing and rear cylinders reduce drag by 22.8% and 7% respectively. The explanation of how the cylinders reduce drag is not well detailed while the fairing reduces drag by improving the airflow over the cabin. Garcia-Ribeiro et al. [88] highlighted the lack of drag reduction devices for commercial buses. In this article, they demonstrated three distinct devices, large vortex generators, lateral side devices with NACA upper surfaces, and roof rails with angled grooves. Together the devices reduce drag by 8.6% with the lateral devices performing the best, as they operate in an area of flow separation near the front of the vehicle. All testing was done in direct flow neglecting how the devices perform in a crosswind. Wang et al. [89] performed a CFD study on a rigid truck outfitting it with a rear boat tail cone and a cab roof fairing to reduce drag, offering a 4.7% and 12.2% saving respectively. The boat tail's length was relatively small but still offered an appreciable drag reduction. Lav [90] outlined the results of a CFD study on a tractor-trailer which used 11 small VGs placed at the rear of the trailer to reduce drag by 9.1%. The baseline $C_{D}$ for the combination was far above that of a realistic vehicle. Only 2 million cells were used on a simplified CAD model. Ortega et al. [91] conducted wind tunnel testing on a tractor-trailer that utilised a base bleed system on the rear of the tractor. The system was proposed as an alternative to side extenders for reducing drag in the tractor-trailer gap. Drag reductions were shown to increase for increasing bleed rate however it was concluded that the savings were not large enough to justify implementing the active system as the power input could potentially outweigh the savings. Shao et al. [92] performed a CFD study on a baseline bus model and replaced its top and bottom rear trailing edges with rotating cylinders to reduce drag. The cylinders rotated such as to oppose the flow and slow it down, reducing its energy upon separation. A 24.7% drag reduction was found at the optimal rotation speeds, which were high and would require considerable device maintenance over time. The authors calculated the power required to rotate the cylinders and concluded it would not outweigh the savings produced. Story et al. [93] performed wind tunnel testing on a vortex trap device fitted within the gap of a tractor-trailer. The device was introduced to the literature by Wood and Bauer [82]. Firstly, it was found that a fully sealed gap reduced wind averaged drag by 20% while an optimised vortex trap device reduced it by 12%. Increasing the length of the device increased the drag reduction. The primary reduction mechanism reduced crossflow and separation during a crosswind while reducing the average pressure on the trailer's face. Mihelic [94] gave a comprehensive overview of methods to improve fuel and freight efficiency for heavy goods vehicles by highlighting past and present work into reducing drag. The work is in line with that published by the North American Council for Freight Efficiency (NACFE). Vallbona [95] detailed a CFD-based report, contracted by DG Clima EU, that provided standard drag reduction values for the application of rear flaps and side covers on a generic tractor and semitrailer. High-fidelity RANS simulations were performed on several different configurations of the devices in both direct and yawed flow. For example, the tall rear flaps and long side covers offered a 5.56% and 5.8% drag reduction in direct flow respectively. Patten et al. [96] outlined an extensive review of the available drag reduction devices for heavy trucks. It noted that in winter in Canada, air density changes can increase drag by nearly 20%. Platooning was found to reduce drag by 9-25% depending on vehicle spacing and speed, base bleeding can reduce drag but lacks practicality over passive techniques, and side skirts were reported to reduce fuel consumption by 3-7%. Boat tails were seen as optimal for drag reduction when sized between 24-32 inches. The review outlines how there is a lack of high-quality peer-reviewed evidence to suggest that VGs can offer significant fuel savings. McAuliffe and Wall [97] used large-scale wind tunnel testing to outline how to maximise the drag savings when using boat tails and side skirts. The features of a boat tail such as length, angles, shape, and the effect of the bottom panel were all studied. Interaction between boat tails and side skirts was beneficial with the combination producing a greater total reduction. The article outlined how boat tail design is a non-linear process and how parameter interactions make creating a set of definitive guidelines difficult.

Hariram et al. [98] provided a good reference for how to improve the aerodynamics of heavy-duty vehicles. Cab devices such as air dams, active grill shutters, and side extenders were discussed along with many more. It noted that OEMs are including many of these devices in their new vehicles, avoiding the need for aftermarket modification. The paper further discussed many of the devices for trailers such as splitter plates, vortex traps, side skirts, and rear tapering panels/boat tails. Several different boat tail designs were mentioned such as filled and unfilled cavities, including cavities with no lower panel. All offering around 4% fuel consumption improvement. Smith et al. [99] detailed a report of when four main North American truck manufacturers came together to test out many of the aerodynamic treatments available for tractor-trailers to improve fuel economy. It was found that by outfitting the vehicles with various aero packages, the results in some cases could offer up to an 11.5% fuel improvement. Miralbes and Castejon [100] studied five different rear devices for heavy vehicles using CFD. Four of the devices were boat tails, all of which provided noticeable drag reduction of between 10-15%. One of the boat tails was uniquely shaped like a water drop and offered an appreciable 13% drag reduction. The other device known as the vortex strake device from [82] was shown to reduce drag by only 0.72%, contradictory to the claimed fuel savings in the original paper. Acevedo-Giraldo et al. [101] looked at the novel topic of reducing drag on a large car carrier when fully loaded with multiple cars. Using CFD to evaluate the performance of five different cover configurations, drag reductions of between 3.9% - 23.8% were reported. The cover that enclosed nearly all of the car carrier's rear performed the best. Fletcher and Stewart [102] performed small-scale wind tunnel testing on a generic bus model, in which modifications were made to the bus's front radii along with adding a novel vortex trap cavity device to the rear. The rear device consisted of an indented section shaped to match the horseshoe vortex that appears on the flat rear of the bus. Together the modifications reduced $C_{D}$ from 0.387 to 0.287. Lee and Lee [103] studied a novel boat tail fitted to the rear of a heavy truck, in which the tail consisted of three conventional top and side sections but included an angled bottom piece that was half the length of the other sections. Using an optimal bottom piece angle of 45°, the device reduced drag by 9% in comparison to 8.2% for a conventional straight four-way tail. The same tail with no bottom piece reduced drag by just 6.5%. PIV was used to show how the tail reduced the size of wake vortices leading to increased base pressure. Altaf et al. [104] outlined a CFD study on a simplified model of a TGX Man truck in which a unique elliptical flap was fitted to the upper trailing edge of the vehicle. Angled at 50° the flap reduced drag by 11.1%, outperforming similar rectangular and triangle-shaped flaps which only achieved reductions of 4.5% - 7%. Additional work on the effects of using perforated flaps was carried out, in which the reductions did not exceed those of the non-perforated designs. The cell count for the simulations was quite low (< 1 million) for such a large test vehicle. Rejniak and Gatto [105] modified the trailing edges of a heavy truck to look like a lobed mixer and compared the reductions to that of a simple 15° tapered rear. The mixer design reduced drag by 7% compared to the 10.8% for the taper, however, the mixer design was a much less invasive modification and did not greatly reduce the trailer's internal volume. Both modifications reduce drag by reducing wake size through enhanced inboard momentum. Mcdonald and Palmer [106] performed a combination of wind tunnel and on-road testing to reduce drag on two common contemporary American buses. Add-on devices included rear splitter plates, rear-projected discs, front turning vanes, and basic front fairings that increased front edge radii. On-road testing of a full front fairing found an 11.7% fuel improvement which matched up well with a 17% wind tunnel drag reduction. In general, the modifications were very primitive and lacked novelty, particularly the front fairings, which effectively just turned the initially blunt bus into a smoother shape seen on most buses today. Landman et al. [107] outlined a high-quality wind tunnel study on a 1/4 scale heavy truck to discover the upper limits for drag reduction using idealised add-on devices. The study was proposed to add context to claims by other authors or companies on the effectiveness of their devices when compared to possible upper limits. The devices studied included a fully sealed gap (7%), a generic full boat tail (10.3%), a practical side skirt (15.7%), an extended side skirt (17.5%), and an impractical full skirt (19.9%). The percentages listed are wind-averaged drag reductions on a baseline truck at 55 mph. Used together the devices reduced drag by 33.4%. The boat tail used in the study was only 0.6 m long when scaled up, hence this did not quantify the device's true upper limit, as longer tails have higher reductions. Ekman et al. [108] took a light-duty truck and modified it using a small boat tail and side skirt, and altered the roof's leading edge combined with a cab roof deflector. With an additional modification that changed the roof's curvature but reduced internal volume, a total 33.6% drag reduction was achieved. Without affecting internal volume a 28.4% reduction was reported. A full-scale prototype was built and operated to validate the CFD results, which found a 12% fuel consumption reduction. Ekman et al. [109] used CFD to model the drag forces on an unloaded timber truck and found that by adding panel shields between the cab and the first stake on the trailer, a 6.7% drag reduction could be achieved. The device discussed was very simple to implement and would not impede daily usage. Articles like this which explore vehicles not commonly studied in the literature are essential if the entire transport network is to reduce its fossil fuel consumption and transition to alternative fuels. McAuliffe et al. [110] explored the aerodynamic effects of modifications to heavy trucks using 30% scale models in a large wind tunnel facility. Removing mirrors reduced drag by 4%. Optimising the shape of the tractor cab reduced drag by between 7-9%. Side skirts reduced drag by around 7% while adding a small boat tail increased the drag reductions with the skirts to 10-11%. When subjected to air from upstream traffic, drag reductions of around 10% were observable, therefore current estimates of transport GHG emissions could be overestimated as vehicles do not always operate in isolated driving conditions.

Törnell et al. [111] focused on the effects of truck-SUV platooning and highlighted how studying unique vehicle combinations is a valid research gap within the literature. Using high-quality CFD and wind tunnel testing, the drag reduction mechanisms were shown to be the same as for truck-truck platooning, in which the trailing vehicle had reduced drag due to slower oncoming flow and the leading vehicle due to an increased base pressure. For optimal positioning, the truck and SUV could have an improved fuel economy of between 2-3% and 30% respectively Törnell et al. [112] evaluated the effectiveness of truck-truck platooning for changing inter-vehicle distance, lateral offset, and yaw angle. Yaw conditions were shown to reduce the efficiency of platooning. The leading vehicle was not very sensitive to a lateral offset while the trailing vehicle was only slightly more sensitive. Drag was shown to reduce for both vehicles as the inter-vehicle distance reduced. McAuliffe et al. [113] described fuel economy testing for three truck platooning on a circular test track to see how various configurations affected drag reduction. The combined effect of platooning when using aero devices utilised a 14.2% fuel saving for the full platoon when separated 17.4 m. The lead vehicle only had small savings of 1% for short distances. The trailing vehicle had the best fuel saving, being 3% higher than that of the middle vehicle. In general, the aero trailer configurations had improved fuel savings when platooning. Buil and Herrer [114] performed a CFD-based study on a vehicle tanker fitted with aerodynamic improvements that reduced drag by a total of 23%. The three main improvements included a side skirt system, a smoothed aerodynamic tank front, and a boat-tailed rear which offered a 9.6%, 6.1%, and 7.7% drag reduction respectively. Tankers are high-drag vehicles and there is a shortage of literature articles detailing how to reduce their drag using appendable devices. Malviya et al. [115] used CFD to model the drag on a simplified high-drag tractor-trailer, which was outfitted with a cab roof fairing and rotating cylinder placed at the upper leading edge of the trailer. The cylinder rotated at 20% of the vehicle's speed and was found to eliminate any flow separation on the leading edge of the trailer. The rotating cylinder reduced baseline drag by 13% which increased to 22% when coupled with the roof fairing. The power to rotate the cylinder was quoted as less than 10% of the total savings realised. The baseline vehicle had an unrealistically high $C_{D}$ of 0.9 due to the tractor having no roof fairing and the trailer's initial leading edge being 90°. Landman et al. [116] performed a full-scale wind tunnel study on a light-duty small truck to reduce drag using appendable devices. These included a front bumper skirt (0.8%), a cone-shaped nose fairing above the cab (7.1%), an 18-inch boat tail (10.9%), a practical side skirt with 8-inches of ground clearance (26.3%) and an ideal skirt with only 3 inches of ground clearance (28.4%). The wind averaged drag reductions for the cumulative addition of each device are outlined in the brackets above for a wind speed of 55 mph. Evidently, side skirts are the most effective device when considering wind-averaged drag. Mosiezny et al. [117] outlined a CFD study on an active flow control device fitted to a utility truck-trailer. The device was applied to the rear edges of the trailer and used a jet of high-speed flow injected ahead of a Coanda surface that helped guide the flow into the wake of the trailer, increasing base pressure. The Coanda surface was shaped like a radial mound on the base of the trailer. Using an optimal flow rate, a maximum drag reduction of over 11% was observed. The same article outlined some initial 2D studies on passive devices, in which an interesting multi-element flap was shown to reduce drag by 12% when angled correctly. Chowdhury et al. [118] highlighted how in certain Asian countries trucks were manufactured with little consideration for aerodynamics. Wind tunnel testing on a baseline Isuzu truck fitted with three different roof fairings was performed, in which the first two were found to increase drag by 33% and 56%. The last fairing was a more appropriate aerodynamic version which reduced drag by 13%. Similar issues are seen in Europe for some utility and livestock trailers, where manufacturers give little consideration to aerodynamics. Future regulation is needed to ensure all vehicle combinations used on roads are appropriately designed to prevent unnecessary drag production. Kehs et al. [119] looked at several different practical boat tail cavities for heavy trucks using both wind tunnel and on-road fuel consumption testing. The best-performing tail reduced drag by 12% and showed a 6.5% fuel saving during the on-road tests. The other tails provided drag reductions of 3-10% with their appearance varying across cavity wall and bottom panel design. The authors highlighted how over the last 30 years many patents of such devices were made only to have little commercial success. The best-performing tail was representative of a product known as the TrailerTail, which has been discontinued. Issues around maintenance and repair costs can affect such a product's commercial success. Kehs et al. [120] performed wind tunnel and on-road testing to see what effect different boat tail cavities have on drag reduction for heavy trucks. It was found that the longer the cavity the greater the drag reduction. Some of the cavities used were inset which may have reduced the maximum reductions that were possible. When tested with side skirts the drag reductions were found to add. On-road testing found fuel savings of 6.5% for the best cavity, which was based on the TrailerTail's design. Seifert et al. [121] demonstrated an active flow device fitted to the rear of a truck with a roller door. Fitted along the rear perimeter, it was shaped like a quarter circle with small holes to facilitate suction and oscillatory blowing at a specified pressure. The device reduced drag by increasing the base pressure on the truck. On-road testing found just a 5% fuel saving after multiple runs, while the conclusion noted that the device did not have a net energy saving due to poor system efficiency. This highlights how implementing efficient active drag reduction devices on real vehicles is difficult. Wood [122] studied five practical drag reduction devices for heavy trucks using wind tunnel testing and extensive fuel economy tests. The devices included a mini skirt (4%), a vortex trap device for the tractor-trailer gap (3%), and a vortex strake device at the end of the trailer (2%). The final two devices were fitted at the rear, one being a straight extension cavity (4%) and a flap device which consisted of two inset side panels on the base (3%). The values outlined relate to fuel economy improvement. Using a combination of the devices a 10% fuel improvement was seen, which related to a 25% drag reduction at 65 mph. Wong et al. [123] used 1/18 scale wind tunnel testing to demonstrate the drag reduction capability of devices for tractor-trailers. The most interesting was a cab roof fairing with slotted air ducts that permitted some flow to move through and hit the trailer face. The slots reduced the amount of flow moving over the cab and helped prevent separation after the fairing. The airflow on the trailer faces was further influenced by guide vanes to help the flow move around sharp corners and reduce separation. Together with a splitter plate mounted in the tractor-trailer gap, the devices realised a 30% drag reduction.

In summary, drag reduction techniques for tractor-trailers is a well-studied and well-refined topic with decades of research already having been conducted. In contrast, similar methods for buses and other heavy transport require further work, as there is a clear need in the literature for more publications and more novel devices. Side skirts could be considered the most effective device for a tractor-trailer, followed by a full rear cavity, and a filler device for the tractor-trailer gap. Cab roof deflectors have become a standard modification on nearly all tractor-trailers. In terms of the rear cavity, a full converging configuration is essential for maximising gains. Removing parts of the cavity such as the bottom or top panel can significantly detriment the overall drag reduction. A big issue with fitting devices to the trailer is there are usually many trailers to one tractor, which increases the payback time for the devices, as a given trailer will have idle time. Lastly, devices designed for heavy transport must consider crosswind effects and aim to optimise the wind-averaged drag, and if possible, consider additional on-road effects such as the influence of traffic.

#### 2.2.3 Vehicle combinations

Kunstner [124] discussed the application of a roof deflector for a passenger car towing a trailer to help guide the flow onto the roof of the trailer. Using large-scale wind tunnel testing a deflector with adjustable position and angle produced an optimum 16.4% drag reduction. The car and trailer geometries are somewhat outdated and the testing did not make use of ground simulation. Hands and Zdravkovich [125] investigated the potential for reducing drag on a car-trailer combination using a flat plate laid down on the drawbar along with a roof deflector fitted to the car's roof. Using small-scale wind tunnel tests it was shown that the best location for the roof deflector was near the rear of the vehicle. The flow field between a trailer and estateback car was considerably better than that for a fastback car and trailer. Atif et al. [126] performed a CFD study on two car-trailer combinations to explore avenues for drag reduction. The cell counts for the CFD models were quite low for what was a large domain and model. The study found that as the drawbar length increases so does the drag, and that 80% of the total drag force was on the trailer. The drag force breakdown is highly dependent on the shape of the trailer and tow vehicle. Adding a top and bottom diffuser to the trailer brought about drag reduction when optimised, but can increase drag for certain diffuser angles. White [127] outlined several projects conducted at Middlesex University on vehicle aerodynamics. Referenced projects found that using a 45-degree car roof deflector reduced car-trailer drag by over 23%, which was in line with values of between 15-30% quoted in the literature at the time. Additionally, work on loaded and unloaded roof racks estimated that drag increased by 26% and 44% respectively. These drag increases were on high-drag cars with CD values of 0.46, hence when fitted to more modern aerodynamic cars the drag increases may be higher. Connolly et al. [128] presented a study on the aerodynamics of flatbed trailers for passenger vehicles and found that by adding various devices to the trailer, significant drag reductions could be realised. When towed by a van, the combination's largest drag contributor was the van's rear. A novel rear-end appendable device known as the multi-stage converging cavity was introduced which reduced drag by 18% and when combined with the trailer devices led to a total 25% drag reduction. The new device consisted of converging boat tail cavities within cavities not previously detailed in the literature.

#### 2.2.4 Vehicle configurations

Taherkhani et al. [129] used a combination of wind tunnel and CFD testing to reduce the drag associated with fitting a light bar to an emergency vehicle. Using an optimised aerofoil-shaped design, drag was reduced by 20%, however, the new light bar took up a lot of roof space which may not be practical. It may have been better to design an appendable drag-reducing device fitted ahead of the standard light bar. Zacharof et al. [130] detailed extensive work by the JRC for the EU Commission into the factors affecting fuel consumption for passenger vehicles. It highlighted the need for more vehicle add-on devices to reduce drag, while reviewing how drag-increasing add-ons like roof racks and roof boxes increase fuel consumption, the degree to which is dependent on the vehicle's shape. The article also addresses the need for more published material on the fuel consumption effects of vehicle add-ons. Lenner [131] detailed the results from on-road fuel economy tests performed with a Volvo sedan. Various add-on components were tested. An empty roof rack was found to increase fuel consumption by 1-3%, a ski box by 10%, a low-profile trailer by 30-50% and a high-profile trailer by 60-80%. It was found that increasing speed from 70 to 90 km/h with the high-profile trailer fitted increased fuel consumption by 25%. This article highlights the need for drag reduction devices when towing trailers. Chen and Meier [132] estimated the total fuel consumption impact for roof racks in the US to be around 1% of the total fuel consumption for light-duty vehicles. It asserted that there is relatively little published material on the fuel consumption penalty for roof racks. The authors outlined the need for a policy that will discourage drivers from driving with unloaded roof racks. Similarly, the need for more aerodynamic roof racks going forward was addressed. Hansen and Blankenship [133] reported the results from fuel consumption testing performed on three different police vehicles using six different lightbar designs. The best-performing light bar increased fuel consumption by around 6.6% while the worst increased it by 11.3%. The average was 8.5%. The study found a much greater fuel efficiency for lower-profile light bars. It was estimated that choosing the lowest drag lightbar would save the department $500,000 over 10 years. Raub [134] outlined the effects of swapping roof-mounted light bars for internal light bars in terms of fuel consumption, accident rates, and officer productivity in a rural setting. The article referenced a previous study conducted in 1982 which found that vehicles without roof light bars had a 6.4% better fuel economy. A new study on 208 vehicles found a 6.9% improved fuel economy for unmarked vehicles using internal lighting. Vehicles without roof-mounted light bars were found less likely to be in accidents. Lastly, there were no significant differences in fuel economy and accidents for vehicles operating in an urban setting due to the lower city-based speeds. Connolly et al. [135] focused on the aerodynamics of the high-drag taxi sign currently in use in Ireland and found that reorientating the sign longitudinally could save drivers approximately €832 a year and reduce national CO2 emissions by 22,464 tonnes annually. Additionally, appendable devices were fitted ahead of the taxi sign in which a smooth ramp was found to dramatically reduce drag. The main cause of drag was the separation zone behind the sign which extended all along the vehicle's roof in some cases. Connolly et al. [136] studied the potential for drag reduction on roof-mounted lightbars on emergency vehicles, with a particular focus on lightbars fitted to police vehicles. The study outlined an optimised rear clip-on device to the standard lightbar when retrofitting that offered a 2.5% drag reduction. Additionally, the study proposed a final optimised lightbar profile for the next generation of lightbar that increased overall vehicle drag by just 2.8%. Studies like this are crucial to fleet managers of police vehicles as they quantify the drag penalties associated with fitting vehicle add-ons, particularly important in the context of fleet electrification with the prospect of range detriment.

### 2.3 Summary of the most effective devices

**Figure 4:**
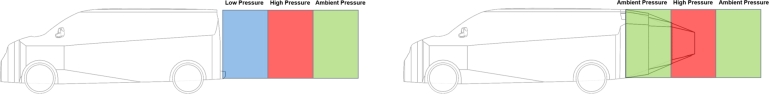
Demonstration of the working principle of the Multi Stage Converging Cavity (Fig. 2v) [128]. The multiple stages allow the base of the vehicle to tap into a high-pressure zone downstream and offset the naturally occurring low-pressure zone on the base of the vehicle, leading to a larger drag reduction than that for a single cavity of the same length.

The key concept to understand is that how effective a device will be is highly dependent on the vehicle type it is fitted to, and how it interacts with the other devices or fittings on that vehicle. Interference effects between devices can sometimes provide much enhanced benefits. Some review articles, such as those discussed in Section 2.4, do provide relative quantification of one device over another in terms of ranking or by providing the expected reduction as a percentage when using that device. This while useful for quantifying the best device, is not entirely effective or correct as there are numerous other factors to take into account. One clear example to outline this, is a rear drag reduction device may report a 20% drag reduction on a given vehicle, however, if that vehicle does not have a smooth underside then the reported drag reduction may be less, as the quoted 20% is dependent on the other devices fitted to the vehicle. This being considered, a general outline of the most effective devices will be given based on all the articles cited in this review. The three primary modifications from least to most effective could be summarised as the devices fitted to or around the wheels of a vehicle, followed by underside covers/trays, followed by rear drag reduction devices. In terms of the devices fitted to the rear, full converging cavities are generally the most effective. The next generation of rear drag reduction devices may include the use of multiple, staged, converging cavities as the reductions outlined when using multiple cavities within one another are much greater than when using just a single cavity. Fig. 4 demonstrates the working principle of the multi stage cavity design. Other rear modifications that consist of individual pieces such as plates, slats, or single top and bottom pieces, generally do not out perform the gains of a well-designed converging full cavity.

### 2.4 Previous review papers

Siddiqui and Agelin-Chaab [137] provided a very comprehensive review of how nature-inspired design can influence bluff body aerodynamics leading to the creation of novel drag reduction devices. Examples included how wavy sections and tubercles could be used on the panels of boat tails. Other opportunities included micro-grooved surfaces that reduce drag like shark riblets, bio-inspired front-end designs, and VGs shaped to match fish fins. Mukut and Abedin [138] presented a review on drag reduction for vehicles. Work outlined included a movable rear diffuser that reduced drag along with how rear blowing and suction applied to the aft of the Ahmed body can reduce drag. Reference was made to plasma actuators which are a novel method of preventing flow separation and can reduce drag by 8% on the Ahmed body but encounter issues when used in rain and winter conditions. Mair [139] provided a review of the drag reduction methods used on 2D bodies such as splitter plates, base bleeding, ventilated cavities, trailing-edge notches, and boat-tailing. It outlined that all the 2D works were not enough to ensure that the methods would work on a real practical 3D vehicle, recommending more testing on 3D bodies. It concluded that base bleeding was the most effective 2D method yet was generally impractical due to the high flow rates required. Additionally, it found that boat tailing was the best technique for axisymmetric bluff bodies, giving appreciable drag reduction up to yaw angles of 15°. Tanner [140] reviewed drag reduction technology for 2D and axisymmetric bodies with a blunt base. It found that when using a rear splitter plate, the thicker the plate the greater the drag reduction when compared to thin plates. It described that changing the splitter plate into a wedge-shaped profile offered a much greater reduction and could substantially reduce the base drag component. It also described how having serrated or segmented trailing edges can give additional drag reduction. Sudin et al. [141] provided a review of the drag reduction methods used on vehicles. Most of the discussion was on active flow control methods with brief descriptions of passive devices such as vortex generators, spoilers, rear diffusers and others. A useful reference table detailing the relevant drag reduction percentages for each of the proposed devices/methods was provided. Altaf et al. [142] presented a short review of passive drag reduction technology. Examples of the devices discussed included works on flaps/deflectors, splitter plates, boat tails, and references to surface modifications such as dimples, tabs, riblets and vents. Szodrai [143] reviewed drag reduction research between 2000-2020, with a primary focus on the methods used for small passenger vehicles. Methods for active and passive drag reduction were discussed, which were then analysed and presented in a table for comparison of maximum expected reductions for specific devices. Overall, this paper gave an excellent account of the drag reduction research discussed in the literature. Eun et al. [144] provided a very brief overview of some of the methods used to reduce drag on bluff bodies. Concepts such as boat tailing, base bleeding, rotating cylinder edges, and splitter plates were discussed, with little detail provided on each. Choi et al. [145] provided a comprehensive review of drag reduction methods for heavy vehicles and outlined an extensive summary of the main devices used on the tractor's cab, the trailer's underside and the vehicle's base. The review highlighted the need for more studies looking at the underbody flow of heavy vehicles, encouraging the proposal of more devices given the underside is responsible for 30% of total vehicle drag. Yu and Bingfu [146] detailed advances in active drag reduction methods on simplified bluff bodies, with an emphasis on how the methods influence rear wake dynamics. The article makes an interesting claim that active methods are more suitable for future implementation as passive techniques may have approached a developmental optimum, and active control methods can have the advantage of being concealable. While automakers do set a premium on methods that preserve external design features to enhance showroom sellability, active methods are expensive to implement and usually require a power input, and in general provide smaller net drag reductions in comparison to optimal passive techniques. Gad-el Hak [147] reviewed the advances in drag reduction coatings up to the early 2000s. The author highlighted how it is a well-studied topic because of its potential applications, and noted how many claims of drag reduction were made through the years only to be later refuted. Considering that mainstream auto manufacturers do not sell cars with such coatings it is a good indication that the tech is not currently viable for road vehicles. The skin friction component for road vehicles can account for only around 10% of total drag [6], making the potential benefit less attractive. Garry [148] provided a short review of drag reduction devices for commercial vehicles in the mid-1980s. The main methods discussed included cab roof fairings, trailer forebody fairings, side skirts, and gap sealers. Operational issues were cited for the more invasive devices.

### 2.5 Patented drag reduction devices

The commercial potential for successful drag reduction devices has led to the filing of many patents for these devices. A search through these patents often shows repetitive designs, and there's a noticeable lack of independent studies validating the effectiveness of these devices in reducing drag as claimed. Due to these factors and for brevity, this paper does not include a review of patented drag reduction devices.

## 3 Outlook and conclusions

Drag reduction has in the past, and still is today, a major goal for efficient vehicle design. What began as a need for vehicle top speed has presently become a priority to realise range extensions for electric vehicles, to reduce fuel expenditure, and to reduce overall GHG emissions for a greener climate. The vision for the future may be that some users will want and require an appendable drag reduction device. The aim is that they will be able to click on and click off, or fold up and deploy it as needed. Issues may arise around how the devices will be mounted to the vehicle, which currently must be done by the user or by the drag reduction device seller. It may be the case that the future demand for such devices increases to the point that manufacturers are mandated to give the option when purchasing a new vehicle to have standard device mounting points fitted, in the same way, a vehicle can be customised for a towing hitch. A user then may have a catalogue of drag reduction devices to choose from, picking the one that best suits their needs. It may be the case that the bigger the device, the greater the potential reduction, but at the cost of added operational overhead and maintenance. The biggest obstacle to drag reduction technology could be seen as device maintenance. Any device that moves or cycles between positions will require maintenance at given intervals. If a device is damaged or involved in an accident on the road or when parking, the costs associated could negate any fuel savings realised through its use. Like with any new technology, there are a lot of things that need to be worked out, and as this review shows, drag reduction devices are far from new and have been around for some time, yet their widespread application on a wide range of modern road vehicles in response to current needs will be new and novel.

The hope is that in the years to come drag reduction research will become more widespread by academic institutions and individuals, assisted by advances in CFD and computing speed. This review has shown that there has been a lot of work done on specific vehicles, such as tractor-trailers, using devices that have been around for some time. Future research should be focused on creating new novel-shaped devices that either offer greatly improved drag reductions or provide appreciable reductions using a smaller, smarter design than the existing state-of-the-art. The literature has in the past been filled with work on simplified bodies, which provide insightful results, yet can fail to work as expected on real road vehicles. Future work should focus on realistic vehicles like the DrivAer model. Another issue present in the literature can be low-quality CAD and CFD, particularly in earlier publications, which thankfully today is becoming less common. Overall, drag reduction research is a well-defined topic, with a rich history, but still requires improvements to represent more road vehicles and to include completely new, novel devices.

## Ethics approval

No ethics approval was required for the work outlined in this review article.

## CRediT authorship contribution statement

**Michael Gerard Connolly:** Writing – review & editing, Writing – original draft, Visualization, Validation, Methodology, Investigation, Funding acquisition, Formal analysis, Data curation, Conceptualization. **Alojz Ivankovic:** Supervision, Software, Resources, Project administration. **Malachy J. O'Rourke:** Supervision, Software, Resources, Project administration.

## Declaration of Competing Interest

The authors declare that they have no known competing financial interests or personal relationships that could have appeared to influence the work reported in this paper.

## Data Availability

No original data was used to create this article. All referenced works are publicly available and can be found under their respective references in the Reference section.

## References

[1] EEA (October 2023). Transport and Mobility. https://www.eea.europa.eu/en/topics/in-depth/transport-and-mobility.

[2] Carlin David (December 2023). Your quick guide to the outcomes of COP28. https://www.forbes.com/sites/davidcarlin/2023/12/13/your-quick-guide-to-the-outcomes-of-cop-28/.

[3] EEA (October 2023). New registrations of electric vehicles in Europe. https://www.eea.europa.eu/en/analysis/indicators/new-registrations-of-electric-vehicles.

[4] Najman Liz (November 2023). EV range anxiety afflicts this group of people the most. https://www.recurrentauto.com/research/ev-range-anxiety-afflicts-this-group-most.

[5] Krishna G. (June 2021). Understanding and identifying barriers to electric vehicle adoption through thematic analysis. Transp. Res. Interdiscip. Perspect..

[6] Schuetz Thomas (2015).

[7] Zhang Chunhui, Bounds Charles Patrick, Foster Lee, Uddin Mesbah (August 2019). Turbulence modeling effects on the CFD predictions of flow over a detailed full-scale sedan vehicle. Fluids.

[8] Aultman Matthew, Wang Zhenyu, Auza-Gutierrez Rodrigo, Duan Lian (March 2022). Evaluation of CFD methodologies for prediction of flows around simplified and complex automotive models. Comput. Fluids.

[9] (November 2019). https://eur-lex.europa.eu/eli/reg/2019/2144/oj/eng.

[10] (March 2021). http://data.europa.eu/eli/reg/2019/2144/oj/eng.

[11] Windsor S.C. (1991). The effect of rear end shape on road vehicle aerodynamic drag. https://trid.trb.org/View/376256.

[12] Ahmed S.R., Ramm G., Faltin G. (1984). Some salient features of the time -averaged ground vehicle wake. SAE Transact..

[13] Storms Bruce L., Ross James C., Heineck James T., Walker Stephen M., Driver David M., Zilliac Gregory G., Bencze Daniel P. (February 2001). https://ntrs.nasa.gov/citations/20010038028.

[14] Nouzawa Takahide, Haruna Shigeru, Hiasa Kazuhiko, Sato Hiroshi (1990). Analysis of wake pattern for reducing aerodynamic drag of notchback model. SAE Transact..

[15] Le Good Geoffrey M., Garry Kevin P. (2004). On the use of reference models in automotive aerodynamics.

[16] Li Ruiying, Borée Jacques, Noack Bernd R., Cordier Laurent, Harambat Fabien (March 2019). Drag reduction mechanisms of a car model at moderate yaw by bi-frequency forcing. Phys. Rev. Fluids.

[17] Hoffmann Felix, Schmidt Hanns-Joachim, Nayeri Christian, Paschereit Oliver (September 2015). Drag reduction using base flaps combined with vortex generators and fluidic oscillators on a bluff body. SAE Int. J. Commer. Veh..

[18] Barros Diogo, Borée Jacques, Noack Bernd R., Spohn Andreas, Ruiz Tony (October 2016). Bluff body drag manipulation using pulsed jets and Coanda effect. J. Fluid Mech..

[19] Howell Jeff, Sheppard Andrew, Blakemore Alex (March 2003). Aerodynamic drag reduction for a simple bluff body using base bleed. 10.4271/2003-01-0995.

[20] N. Yurchenko, Thermal riblets: conceptual approach to flow control, 2015, pp. 35–37.

[21] Zhang B.F., Liu K., Zhou Y., To S., Tu J.Y. (December 2018). Active drag reduction of a high-drag Ahmed body based on steady blowing. J. Fluid Mech..

[22] Geropp D., Odenthal H.-J. (January 2000). Drag reduction of motor vehicles by active flow control using the Coanda effect. Exp. Fluids.

[23] Gagnon Louis, Richard Marc J., Lévesque Benoît (June 2011). Simulation of a rotating device that reduces the aerodynamic drag of an automobile. Trans. Can. Soc. Mech. Eng..

[24] Lorite-Díez M., Jiménez-González J.I., Pastur L., Cadot O., Martínez-Bazán C. (May 2020). Drag reduction on a three-dimensional blunt body with different rear cavities under cross-wind conditions. J. Wind Eng. Ind. Aerodyn..

[25] Garcia De La Cruz J. Marcos, Brackston Rowan D., Morrison Jonathan F. (August 2017). Adaptive base-flaps under variable cross-wind. 10.4271/2017-01-7000.

[26] Wong D.T.-M., Mair W.A. (July 1983). Boat-tailed afterbodies of square section as drag-reduction devices. J. Wind Eng. Ind. Aerodyn..

[27] Lorite-Díez M., Jiménez-González J.I., Gutiérrez-Montes C., Martínez-Bazán C. (October 2017). Drag reduction of slender blunt-based bodies using optimized rear cavities. J. Fluids Struct..

[28] Gilliéron Patrick, Kourta Azeddine (January 2010). Aerodynamic drag reduction by vertical splitter plates. Exp. Fluids.

[29] Park Hyungmin, Lee Dongkon, Jeon Woo-Pyung, Hahn Seonghyeon, Kim Jeonglae, Kim Jungwoo, Choi Jin, Choi Haecheon (September 2006). Drag reduction in flow over a two-dimensional bluff body with a blunt trailing edge using a new passive device. J. Fluid Mech..

[30] Hassaan Mohab, Badlani Divyang, Nazarinia Mehdi (December 2018). On the effect of boat-tails on a simplified heavy vehicle geometry under crosswinds. J. Wind Eng. Ind. Aerodyn..

[31] Howell Jeff, Sims-Williams David, Sprot Adam, Hamlin Fred, Dominy Robert (April 2012). Bluff body drag reduction with ventilated base cavities. SAE Int. J. Passeng. Cars, Mech. Syst..

[32] Hirst Trevor, Li Chuanpeng, Yang Yunchao, Brands Eric, Zha Gecheng (September 2015). Bluff body drag reduction using passive flow control of jet boat tail. SAE Int. J. Commer. Veh..

[33] Pujals G., Depardon S., Cossu C. (November 2010). Drag reduction of a 3D bluff body using coherent streamwise streaks. Exp. Fluids.

[34] Darabasz T., Bonnavion G., Cadot O., Goraguer Y., Borée J. (January 2023). Drag reduction using longitudinal vortices on a flat-back Ahmed body. Exp. Fluids.

[35] Beaudoin Jean-François, Aider Jean-Luc (April 2008). Drag and lift reduction of a 3D bluff body using flaps. Exp. Fluids.

[36] Kowata Shinsuke, Ha Jongsoo, Yoshioka Shuya, Kato Takuma, Kohama Yasuaki (October 2008). Drag force reduction of a bluff-body with an underbody slant and rear flaps. SAE Int. J. Commer. Veh..

[37] Verzicco R., Fatica M., Iaccarino G., Moin P., Khalighi B. (December 2002). Large eddy simulation of a road vehicle with drag-reduction devices. AIAA J..

[38] Robertson Iain, Becot Adrien, Gaylard Adrian, Thornber Ben (May 2014). Automotive drag reduction through distributed base roughness elements. Appl. Mech. Mater..

[39] Siddiqui Naseeb Ahmed, Agelin-Chaab Martin (October 2022). Experimental investigation of the flow features around an elliptical Ahmed body. Phys. Fluids.

[40] Aranha R., Siddiqui N.A., Pao W.Y., Agelin-Chaab M. (May 2023). Development of an intelligent passive device generator for road vehicle applications. J. Appl. Fluid Mech..

[41] Khalighi Bahram, Chen Kuo-Huey, Iaccarino Gianluca (July 2012). Volume 1: Symposia, Parts A and B.

[42] Aider Jean-Luc, Beaudoin Jean-François, Wesfreid José Eduardo (May 2010). Drag and lift reduction of a 3D bluff-body using active vortex generators. Exp. Fluids.

[43] Lajos Tamás (June 1986). Drag reduction by the production of a separation bubble on the front of a bluff body. J. Wind Eng. Ind. Aerodyn..

[44] Capone Alessandro, Romano Giovanni Paolo (February 2019). Investigation on the effect of horizontal and vertical deflectors on the near-wake of a square-back car model. J. Wind Eng. Ind. Aerodyn..

[45] Nabutola Kaloki L., Boetcher Sandra K.S. (December 2021). Assessment of conventional and air-jet wheel deflectors for drag reduction of the DrivAer model. Adv. Aerodyn..

[46] Ferraris Alessandro, De Carvalho Pinheiro Henrique, Giancarlo Airale Andrea, Carello Massimiliana, Berti Polato Davide (March 2021). City car drag reduction by means of flow control devices. 10.4271/2020-36-0080.

[47] Yang Zhendong, Jin Yifeng, Gu Zhengqi (October 2021). Aerodynamic shape optimization method of non-smooth surfaces for aerodynamic drag reduction on a minivan. Fluids.

[48] Magnus Urquhart, Varney Max, Sebben Simone, Passmore Martin (December 2021). Drag reduction mechanisms on a generic square-back vehicle using an optimised yaw-insensitive base cavity. Exp. Fluids.

[49] Datta Basudev, Goel Vaibhav, Garg Shivam, Singh Inderpreet (2019). Study of various passive drag reduction techniques on external vehicle aerodynamics performance: CFD based approach.

[50] Selvaraju P.N., Satish Kumar D. (2022). Effect of external devices on aerodynamic performance of SUV car model. Mater. Today Proc..

[51] Irving Brown Y.A., Windsor S., Gaylard A.P. (April 2010). The effect of base bleed and rear cavities on the drag of an SUV. 10.4271/2010-01-0512.

[52] Vdovin Alexey, Bonitz Sabine, Landstrom Christoffer, Lofdahl Lennart (April 2013). Investigation of wheel ventilation-drag using a modular wheel design concept. SAE Int. J. Passeng. Cars, Mech. Syst..

[53] Varney Max, Passmore Martin, Gaylard Adrian (March 2017). The effect of passive base ventilation on the aerodynamic drag of a generic SUV vehicle. SAE Int. J. Passeng. Cars, Mech. Syst..

[54] De Souza Fenella, Raeesi Arash, Belzile Marc, Caffrey Cheryl, Schmitt Andreas (April 2019). Investigation of drag reduction technologies for light-duty vehicles using surface, wake and underbody pressure measurements to complement aerodynamic drag measurements. SAE Int. J. Adv. Curr. Pract. Mobil..

[55] Kim Inchul, Chen Hualei, Shulze Roger C. (April 2006). A rear spoiler of a new type that reduces the aerodynamic forces on a mini-van. 10.4271/2006-01-1631.

[56] Ranjan Paul Akshoy, Jain Anuj, Alam Firoz (April 2019). Drag reduction of a passenger car using flow control techniques. Int. J. Automot. Technol..

[57] Howell Jeff, Varney Max, Passmore Martin, Butcher Daniel (February 2023). The aerodynamic effects of a 3D streamlined tail on the windsor body. Fluids.

[58] Nath Devang S., Chandra Pujari Prashant, Jain Amit, Rastogi Vikas (December 2021). Drag reduction by application of aerodynamic devices in a race car. Adv. Aerodyn..

[59] Hoerner Sighard F. (1965).

[60] Ilea L., Iozsa D. (November 2018). Wheels aerodynamics and impact on passenger vehicles drag coefficient. IOP Conf. Ser., Mater. Sci. Eng..

[61] Fukuda Hitoshi, Yanagimoto Kazuo, China Hiroshi, Nakagawa Kunio (1995). Improvement of vehicle aerodynamics by wake control. JSAE Rev..

[62] Koike Masaru, Nagayoshi Tsunehisa, Hamamoto Naoki (2004).

[63] Urquhart Magnus, Sebben Simone (August 2022). Optimisation of trailing edge flaps on the base cavity of a vehicle for improved performance at yaw. Flow Turbul. Combust..

[64] Urquhart Magnus, Sebben Simone, Sterken Lennert (August 2018). Numerical analysis of a vehicle wake with tapered rear extensions under yaw conditions. J. Wind Eng. Ind. Aerodyn..

[65] Sebben Simone, Sterken Lennert, Wölken Thies (August 2017). Characterization of the rear wake of a SUV with extensions and without extensions. Proc. Inst. Mech. Eng., Part D, J. Automob. Eng..

[66] Sterken Lennert, Lofdahl Lennart, Sebben Simone, Walker Tim (April 2014). Effect of rear-end extensions on the aerodynamic forces of an SUV. 10.4271/2014-01-0602.

[67] Kang Seung-On, Cho Jun-Ho, Jun Sang-Ook, Park Hoon-Il, Song Ki-Sun, Kee Jung-Do, Kim Kyu-Hong, Lee Dong-Ho (April 2012). A study of an active rear diffuser device for aerodynamic drag reduction of automobiles. 10.4271/2012-01-0173.

[68] Cho Junho, Kim Tae-Kyung, Kim Kyu-Hong, Yee Kwanjung (December 2017). Comparative investigation on the aerodynamic effects of combined use of underbody drag reduction devices applied to real sedan. Int. J. Automot. Technol..

[69] Cho Junho, Park Joonmin, Yee Kwanjung, Kim Hak-Lim (July 2018). Comparison of various drag reduction devices and their aerodynamic effects on the DrivAer model. SAE Int. J. Passeng. Cars, Mech. Syst..

[70] Morelli Alberto (March 2000). A new aerodynamic approach to advanced automobile basic shapes. 10.4271/2000-01-0491.

[71] Peterson R.L. (August 1981). https://ntrs.nasa.gov/citations/19810020556.

[72] McNamara Kathleen M., Jacob Jamey D., Loh Ben, Tsuruta Ryohei, Tsukada Taro, Itakura Eiji, Gandhi Umesh (June 2018). 2018 Applied Aerodynamics Conference.

[73] Chen Kuo-Huey, Khalighi Bahram (April 2015). A CFD study of drag reduction devices for a full size production pickup truck. 10.4271/2015-01-1541.

[74] Taniguchi Keiichi, Shibata Akiyoshi, Murakami Mikako, Oshima Munehiko (March 2017). A study of drag reduction devices for production pick-up trucks. 10.4271/2017-01-1531.

[75] Sirenko Volodymyr, Pavlovs'ky Roman, Rohatgi Upendra S. (July 2012). Volume 1: Symposia, Parts A and B.

[76] Ohno Hideaki, Kohri I. (1991). Improvement of aerodynamic characteristics of a passenger car by side–airdams. Int. J. Veh. Des..

[77] Hucho W.H., Janssen L.J., Emmelmann H.J. (February 1976). https://www.sae.org/publications/technical-papers/content/760185/.

[78] Pamadi Bandu N. (1990).

[79] Maxwell Timothy T., Jones Jesse C., Jones William B. (October 1988). Pickup truck drag reduction-devices that reduce drag without limiting truck utility. 10.4271/881874.

[80] Carr G.W. (February 1976). https://www.sae.org/publications/technical-papers/content/760187/.

[81] Buchheim R., Deutenbach K.-R., Lückoff H.-J. (February 1981). Necessity and premises for reducing the aerodynamic drag of future passenger cars. 10.4271/810185.

[82] Wood Richard M., Bauer Steven X.S. (November 2003). Simple and low-cost aerodynamic drag reduction devices for tractor-trailer trucks. 10.4271/2003-01-3377.

[83] Cooper Kevin R. (November 2003). Truck aerodynamics reborn - lessons from the past. 10.4271/2003-01-3376.

[84] Englar Robert J. (May 2001). Advanced aerodynamic devices to improve the performance, economics, handling and safety of heavy vehicles. 10.4271/2001-01-2072.

[85] Van Raemdonck G.M.R., Van Tooren M.J.L., Dillmann Andreas, Orellano Alexander (2016). The Aerodynamics of Heavy Vehicles III.

[86] Bayindirli Cihan (December 2021). Reducing of pressure based drag force of a bus model by flow control rod in wind tunnel. Int. J. Automot. Sci. Technol..

[87] Yang Xiaolong, Ma Zihui, Li Kenli, Xiao Zheng, Wang Yan, Du Jiayi, Li Keqin (2014). Parallel Computational Fluid Dynamics.

[88] Garcia-Ribeiro Daniel, Bravo-Mosquera Pedro D., Ayala-Zuluaga Jimmy A., Martinez-Castañeda Diego F., Valbuena-Aguilera Jeisson S., Cerón-Muñoz Hernán D., Vaca-Rios John J. (June 2023). Drag reduction of a commercial bus with add-on aerodynamic devices. Proc. Inst. Mech. Eng., Part D, J. Automob. Eng..

[89] Wang Dong, Wang Yansong, Han Yu, Dang Yan, Fan Dengyun, Li Liguang, Society of Automotive Engineers of China (SAE-China) (2015). Proceedings of SAE-China Congress 2014: Selected Papers.

[90] Lav Chitrarth (September 2013). Three dimensional CFD analysis on aerodynamic drag reduction of a bluff tractor trailer body using vortex generators. 10.4271/2013-01-2458.

[91] Ortega Jason, Salari Kambiz, Storms Bruce, Browand Fred, McCallen Rose, Ross James (2009). The Aerodynamics of Heavy Vehicles II: Trucks, Buses, and Trains.

[92] Shao Nan, Yao Guofeng, Zhang Chang, Wang Min (2017). A new method to optimize the wake flow of a vehicle: the leading edge rotating cylinder. Math. Probl. Eng..

[93] Story Joseph, Vallina Garcia Isabel, Babinsky Holger (March 2021). The effect of cross-flow vortex trap devices on the aerodynamic drag of road haulage vehicles. 10.4271/2021-01-5040.

[94] Mihelic Rick (September 2016). Fuel and freight efficiency - past, present and future perspectives. SAE Int. J. Commer. Veh..

[95] Vallbona Albert Gascón (July 2023). https://climate.ec.europa.eu/system/files/2023-07/policy_transport_road_co2_report_20230726_en.pdf.

[96] Patten Jeff, Mcauliffe Brian, Mayda William, Tanguay Bernard (May 2012). https://tc.canada.ca/sites/default/files/migrated/aerodynamics_report_may_2012.pdf.

[97] McAuliffe Brian R., Wall Alanna S. (September 2016). Aerodynamic performance of flat-panel boat-tails and their interactive benefits with side-skirts. SAE Int. J. Commer. Veh..

[98] Hariram Adithya, Koch Thorsten, Mårdberg Björn, Kyncl Jan (October 2019). A study in options to improve aerodynamic profile of heavy-duty vehicles in Europe. Sustainability.

[99] Smith Scott, Younessi Karla, Markstaller Matt, Schlesinger Dan, Bhatnagar Bhaskar, Smith Donald, Banceu Bruno, Schoon Ron, Sharma V.K., Kachmarsky Mark (April 2007). http://www.osti.gov/servlets/purl/926158-qChKeT/.

[100] Miralbes R., Castejon L. (2012). Aerodynamic analysis of some boat tails for heavy vehicles. Int. J. Heavy Veh. Syst..

[101] Acevedo-Giraldo Daniel, Botero-Bolivar Laura, Munera-Palacio Daniel, García-Navarro Juan Guillermo (September 2018). Aerodynamic evaluation of different car carrier devices for drag reduction using CFD. J. Aerosp. Technol. Manag..

[102] Fletcher C.A.J., Stewart G.D.H. (September 1986). Bus drag reduction by the trapped vortex concept for a single bus and two buses in tandem. J. Wind Eng. Ind. Aerodyn..

[103] Lee Eui Jae, Lee Sang Joon (November 2017). Drag reduction of a heavy vehicle using a modified boat tail with lower inclined air deflector. J. Vis..

[104] Altaf Alamaan, Omar Ashraf A., Asrar Waqar (November 2014). Passive drag reduction of square back road vehicles. J. Wind Eng. Ind. Aerodyn..

[105] Rejniak Aleksandra Anna, Gatto Alvin (June 2022). On the drag reduction of road vehicles with trailing edge-integrated lobed mixers. Proc. Inst. Mech. Eng., Part D, J. Automob. Eng..

[106] Mcdonald Alan T., Palmer George M. (November 1980). Aerodynamic drag reduction of intercity buses. 10.4271/801404.

[107] Landman Drew, Wood Richard, Seay Whitney, Bledsoe John (October 2009). Understanding practical limits to heavy truck drag reduction. SAE Int. J. Commer. Veh..

[108] Ekman Petter, Gardhagen Roland, Virdung Torbjorn, Karlsson Matts (April 2016). Aerodynamic drag reduction of a light truck - from conceptual design to full scale road tests. 10.4271/2016-01-1594.

[109] Ekman Petter, Gardhagen Roland, Virdung Torbjörn, Karlsson Matts (2016). Aerodynamics of an unloaded timber truck-a CFD investigation. SAE Int. J. Commer. Veh..

[110] McAuliffe Brian R., Ghorbanishohrat Faegheh, Barber Hali (July 2022). https://nrc-publications.canada.ca/eng/view/object/?id=610b10b1-805a-4047-a908-174b18a0ea07.

[111] Törnell Johannes, Sebben Simone, Elofsson Per (October 2023). Effects of separation distance, lateral offset, and yaw on the drag of a truck and SUV platoon. J. Wind Eng. Ind. Aerodyn..

[112] Törnell Johannes, Sebben Simone, Elofsson Per (June 2021). Experimental investigation of a two-truck platoon considering inter-vehicle distance, lateral offset and yaw. J. Wind Eng. Ind. Aerodyn..

[113] McAuliffe Brian R., Croken Mark, Ahmadi-Baloutaki Mojtaba, Raeesi Arash (April 2017). https://nrc-publications.canada.ca/eng/view/object/?id=d21e1097-5d30-4a0f-b742-35ffad931c2f.

[114] Buil Ramon Miralbes, Castejon Herrer Luis (April 2009). Aerodynamic analysis of a vehicle tanker. J. Fluids Eng..

[115] Malviya V., Mishra R., Fieldhouse J. (January 2009). CFD investigation of a novel fuel-saving device for articulated tractor-trailer combinations. Eng. Appl. Comput. Fluid Mech..

[116] Landman Drew, Cragun Matthew, McCormick Mike, Wood Richard (September 2011). Drag reduction of a modern straight truck. SAE Int. J. Commer. Veh..

[117] Mosiezny Jedrzej, Ziegler Bartosz, Grzymislawski Przemyslaw, Slefarski Rafal (2020). Base drag reduction concept for commercial road vehicles. Energy.

[118] Chowdhury Harun, Loganathan Bavin, Mustary Israt, Moria Hazim, Alam Firoz (March 2017). Effect of various deflectors on drag reduction for trucks. Energy Proc..

[119] Kehs J., Visser K., Grossmann J., Horrell C., Smith A., Dillmann Andreas, Orellano Alexander (2016). The Aerodynamics of Heavy Vehicles III.

[120] Kehs Joshua P., Visser Kenneth D., Grossman Jeff, Niemiec Jared, Smith Andrew, Horrell Charles M. (September 2013). A comparison of full scale aft cavity drag reduction concepts with equivalent wind tunnel test results. SAE Int. J. Commer. Veh..

[121] Seifert A., Dayan I., Horrell C., Grossmann J., Smith A., Dillmann Andreas, Orellano Alexander (2016). The Aerodynamics of Heavy Vehicles III.

[122] Wood Richard M. (2008). Operationally-practical & aerodynamically-robust heavy truck trailer drag reduction technology. SAE Int. J. Commer. Veh..

[123] Wong H.Y., Cox R.N., Rajan A. (November 1981). Drag reduction of trailer-tractor configuration by aerodynamic means. J. Wind Eng. Ind. Aerodyn..

[124] Kunstner R. (1985). Aerodynamic investigations on passenger car/camping-trailer combinations. ATZ, Automobiltech. Z..

[125] Hands S.J., Zdravkovich M.M. (November 1981). Drag reduction for a passenger car towing a caravan. J. Wind Eng. Ind. Aerodyn..

[126] Atif Muhammad, Aliyu Aliyu, Mishra Rakesh (February 2022). Aerodynamic analysis of a car-trailer combination using CFD based numerical techniques: a shape optimisation and drag reduction study. Int. J. COMADEM.

[127] White A.S. (January 1999). Twenty years of projects on vehicle aerodynamics. Int. J. Mech. Eng. Educ..

[128] Gerard Connolly Michael, O'Rourke Malachy J., Ivankovic Alojz (November 2023). Reducing aerodynamic drag on flatbed trailers for passenger vehicles using novel appendable devices. Fluids.

[129] Taherkhani A.R., de Boer G.N., Gaskell P.H., Gilkeson C.A., Hewson R.W., Keech A., Thompson H.M., Toropov V.V. (2015). Aerodynamic drag reduction of emergency response vehicles. Adv. Automob. Eng..

[130] Zacharof Nikiforos-Georgios, Fontaras Georgios, Ciuffo Biagio, Tsiakmakis Stefanos, Anagnostopoulos K., Marotta A., Pavlovic J. (2016). https://data.europa.eu/doi/10.2790/140640.

[131] Lenner Magnus (February 1998). 10.4271/980682.

[132] Chen Yuche, Meier Alan (May 2016). Fuel consumption impacts of auto roof racks. Energy Policy.

[133] Hansen Jack H., Blankenship Joe L. (1986). Highway patrol light bar effects on vehicle fuel efficiency. Transp. Res. Rec..

[134] Raub Richard A. (1985). Removal of roof-mounted emergency lighting from police patrol vehicles: an evaluation. Transp. Res. Rec..

[135] Gerard Connolly Michael, O'Rourke Malachy J., Ivankovic Alojz (September 2023). A drag reduction study on the aerodynamics of the Irish taxi sign. Fluids.

[136] Gerard Connolly Michael, O'Rourke Malachy J., Ivankovic Alojz (May 2024). Reducing aerodynamic drag on roof-mounted lightbars for emergency vehicles. Fluids.

[137] Siddiqui Naseeb Ahmed, Agelin-Chaab Martin (June 2021). Nature-inspired solutions to bluff body aerodynamic problems: a review. J. Mech. Eng. Sci..

[138] Mominul A.N.M., Mukut Islam, Zoynal Abedin Mohammad (March 2019). Review on aerodynamic drag reduction of vehicles. Int. J. Eng. Mater. Manuf..

[139] Mair W.A., Sovran Gino, Morel Thomas, Mason William T. (1978). Aerodynamic Drag Mechanisms of Bluff Bodies and Road Vehicles.

[140] Tanner M. (January 1975). Reduction of base drag. Prog. Aerosp. Sci..

[141] Nizam Sudin Mohd, Azman Abdullah Mohd, Anuar Shamsuddin Shamsul, Ramli Faiz Redza, Mohd Musthafah (2014). Rev. Res. Veh. Aerodyn. Drag Reduct. Meth..

[142] Altaf Alaman, Omar Ashraf A., Asrar Waqar (May 2014). Review of passive drag reduction techniques for bluff road vehicles. IIUM Eng. J..

[143] Szodrai Ferenc (June 2020). Quantitative analysis of drag reduction methods for blunt shaped automobiles. Appl. Sci..

[144] Chuan Eun L., Shakrine Mohd Rafie A., Wiriadidjaja Surjatin, Faruqi Marzuki O. (2018). An overview of passive and active drag reduction methods for bluff body of road vehicles. Int. J. Eng. Technol..

[145] Choi Haecheon, Lee Jungil, Park Hyungmin (January 2014). Aerodynamics of heavy vehicles. Annu. Rev. Fluid Mech..

[146] Yu Zhou, Bingfu Zhang (January 2022). Recent advances in wake dynamics and active drag reduction of simple automotive bodies. Appl. Mech. Rev..

[147] Hak Mohamed Gad-el (January 2002). Compliant coatings for drag reduction. Prog. Aerosp. Sci..

[148] Garry K.P. (July 1985). A review of commercial vehicle aerodynamic drag reduction techniques. Proc. Inst. Mech. Eng., Part D, Transp. Eng..

